# Cerebral cavernous malformations in pregnancy: A systematic review of case reports and case series of hemorrhagic risk and outcomes

**DOI:** 10.1007/s10143-026-04136-w

**Published:** 2026-01-30

**Authors:** Matteo Palermo, Alessio Albanese, Francesco Doglietto, Alessandro Olivi, Carmelo Lucio Sturiale

**Affiliations:** Department of Neurosurgery, Fondazione Policlinico Universitario A. Gemelli IRCCS, Università Cattolica del Sacro Cuore, Rome, Italy

**Keywords:** Cavernoma, Cavernous malformation, Cavernous angioma, Pregnancy, Partum, Puerpuerium

## Abstract

Cavernous malformations (CMs) are vascular lesions that can lead to seizures or hemorrhage. Although pregnancy involves hormonal and circulatory changes that might influence CM behavior, whether it increases the risk of bleeding is unclear. We performed a systematic review using PubMed/MEDLINE and Scopus of published reports describing pregnant women diagnosed with cerebral or spinal CMs. The study question was framed using the PEO strategy, with pregnant women with cavernous angioma as the population, hemorrhage as the exposure, and clinical outcomes as the endpoints. The search was updated to July 12th, 2025 with no time restrictions. This review followed the PRISMA 2020 guidelines. Thirty-two studies were selected accounting for 94 patients. Most lesions were localized in the brainstem and supratentorial regions. Among patients that were symptomatic during gestation, hemorrhage occurred in 70.9% and seizures in 34.1%. However, a secondary analysis limited to comparative studies consistently showed a low hemorrhage rate during pregnancy, ranging from 0.9% to 3%. Most patients were managed conservatively. Surgical intervention was reserved for cases with acute neurological deterioration or persistent hemorrhage, located below the tentorium. Although, more on prudence than evidence, cesarean delivery was frequently chosen. The secondary analysis on the larger cohorts confirms that pregnancy does not increase the risk of bleeding. Treatment, however, must be postponed after delivery. Nonetheless, in cases where early intervention is unavoidable, due to topography and severity of maternal symptoms, surgery might be an option. Additionally, close monitoring in the early postpartum period remains indispensable.

**Supplementary Information:**

The online version contains supplementary material available at 10.1007/s10143-026-04136-w.

## Introduction

Cavernous malformations (CMs), also called cavernomas or cavernous angiomas, are vascular lesions consisting of clusters of abnormally dilated capillary channels without intervening neural tissue. These low-pressure, low‐flow lesions often develop surrounding hemosiderin from repeated microhemorrhages. CMs affect roughly 0.4–0.8% of the population, accounting for about 10–25% of all intracranial vascular malformations [11, 22, 33, 36]. Most are supratentorial, with the remainder in the posterior fossa or spinal cord. Clinically, many CMs are silent and found incidentally on MRI, but symptomatic lesions can present with seizures or with focal neurologic deficits [36].

Pregnancy induces profound hormonal and vascular changes that could affect CM behavior. Elevated levels of estrogen, progesterone, and angiogenic growth factors during gestation might theoretically promote CM growth or bleeding. Historically, some case studies suggested that pregnancy “predisposes women to hemorrhage” from CMs, instead, more recent data have been reporting that the CM hemorrhage rate during pregnancy appears similar to the nonpregnant baseline [16, 19, 36].

The mode of delivery is also debated and still represent an issue: while vaginal delivery is not absolutely contraindicated, many specialists elect cesarean section to avoid the blood pressure spikes and Valsalva strain of labor that could theoretically precipitate rebleeding. Moreover, there is no clear consensus regarding the optimal management strategy when cavernous malformations become symptomatic during pregnancy, particularly in cases presenting with seizures. Therefore, it is clear that there are no rigid guidelines for CMs in pregnancy and decisions remain individualized, relying on the severity of neurological presentation, gestational age, and a multidisciplinary obstetric-neurosurgical approach [16, 19, 36].

This systematic review aims to gather and summarize all available evidence on how cavernous malformations present and are managed during pregnancy. Our goal is to describe how often bleeding and seizures occur, where these lesions are most commonly located, how they are treated, and what outcomes have been reported for mothers.

## Methods

**Fig. 1:**
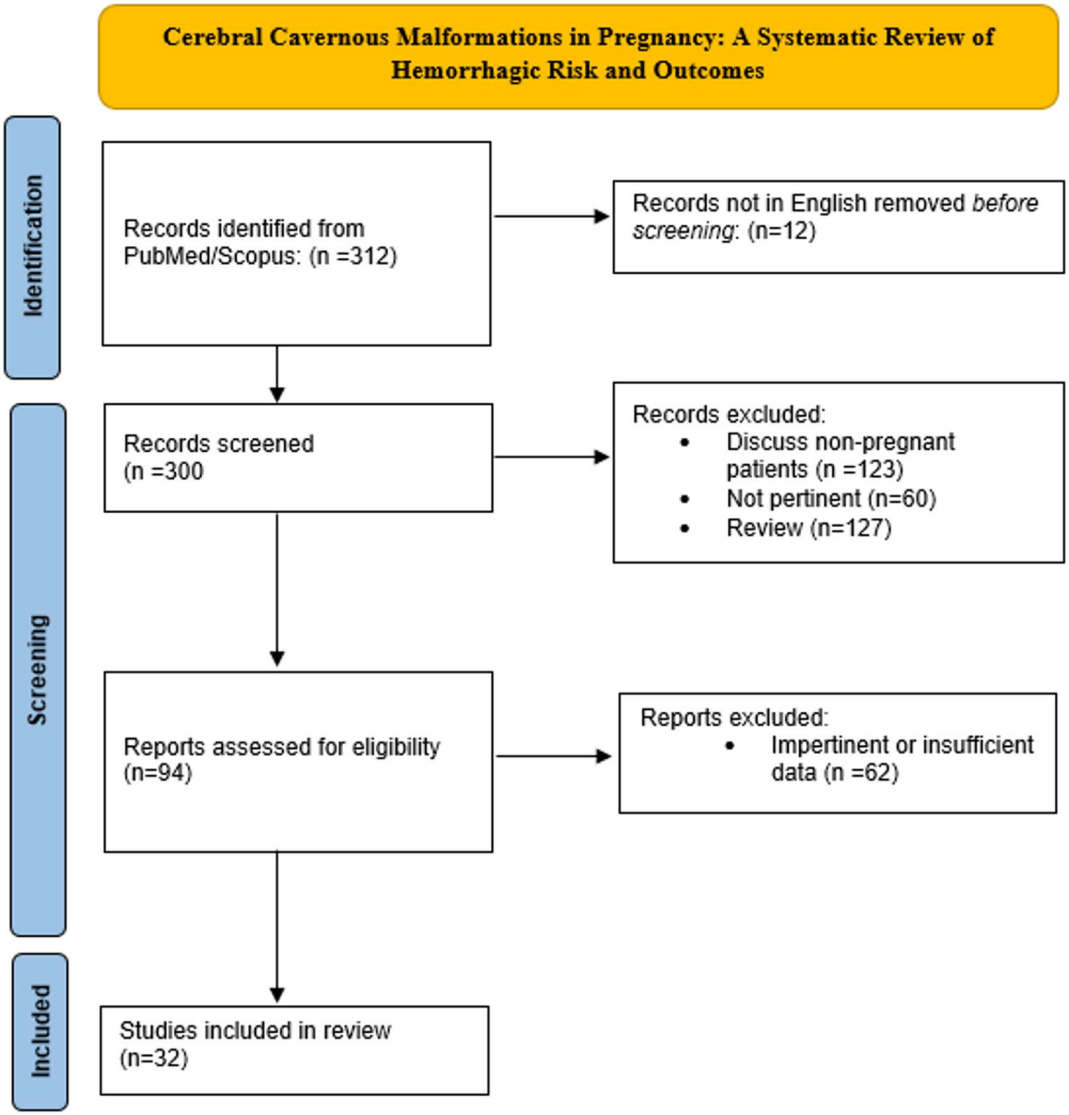
PRISMA flowchart summarizing the study selection process

This review followed the PRISMA 2020 guidelines [24]. To frame the study question, we adopted the PEO strategy: the population of interest was pregnant women with cavernous angioma; the exposure of concern was hemorrhage; and the outcomes evaluated were clinical consequences (Fig. 1).

### Search methods

A comprehensive search on PubMed/MEDLINE, Scopus and EMBASE databases was performed by two authors (CLS and MP) to retrieve relevant studies reporting on gravid patients with a diagnosis of cavernous angioma during the course of gestation: “(cavernoma OR cavernous angioma OR cavernous hemangioma OR cavernous malformation) AND (pregnancy OR parturition OR puerperium OR pregnant OR pregn*)”. The literature search was updated through July 12, 2025, without time restrictions. To enhance coverage, reference lists of the included articles were also screened.

### Selection of studies

Our inclusion criteria were papers describing pregnant patients with a newly diagnosed or previously known cavernoma. Only patients who underwent MRI or had histopathological confirmation of the lesion after surgery were included. This criterion was applied because, in sporadic spinal cases, hemorrhagic tumors or other pathological entities cannot be reliably distinguished on standard CT or angiographic imaging. Animal or pre-clinical studies, review articles, and papers lacking explicit data were excluded. We restricted the search to peer-reviewed publications in English that contained quantitative data. The screening of titles and abstracts was independently performed by two authors (CLS and MP) to determine the inclusion. Followed the full-text screening of the remaining studies to confirm eligibility based on the inclusion criteria (Fig. 1). Re-reading of the articles and joint re-evaluation of the extracted data was performed in case of disagreements. Attempts were made to obtain individual participant data from larger studies to reduce potential bias; however, data sharing was not possible.

### Data extraction

For each eligible study, we extracted and summarized the relevant data in Table 1, including the author, year of publication, and the number of patients reported. In addition, we gathered data on maternal characteristics, such as age, parity, and comorbidities. With respect to the cavernous angiomas, we retrieved information regarding familiality, Zabramski grade, lesion location, and the gestational age (GA) at the time of symptoms onset. In all sporadic cases, the lesion was detected through imaging performed at the time of symptom onset, whereas in familial cases, the reported “GA presentation” refers exclusively to the onset of clinical manifestation of CMs that had already been diagnosed prior to pregnancy. This is because no cases of delayed diagnosis of familiar CMs were reported. We also recorded details on clinical presentation, management during pregnancy, and in case of surgical resection, we specified the gestational age at the time of the intervention. In case of surgical or pharmacological management during gestation, patient-related treatment outcomes were not retrievable. Additionally, all surgical cases involved procedures addressing both the intracerebral hemorrhage and the underlying cavernous malformation itself. Furthermore, we assessed gestational outcomes by documenting the mode of delivery and fetal status. Maternal outcomes were not reported in the included studies.

For the three large case-series, only individual patients with hemorrhagic presentation were detailed in the studies; therefore, these cases are summarized in Table 1 [16, 17, 36]. Instead, the comparative case-control results across these three studies are presented in Table 2.

**Table 1:** Patients with Cavernoma during pregnancy

Author, Year	*N*° patients	Maternal Age	Parity (*N*/M)	Diagnostic tool	Sporadic/Familiar (S/F)	Comorbidities	Zabramski	Site of CM	GA presentation	Onset of Haemorrhage	Onset of Seizures	Focal neurological sign	Headache	Treatment during pregnancy	Excision	GA at delivery	Mode of delivery
Ondra,1988	1	23	M	MRI	S	N/A	N/A	Pons and Midbrain	N/A	X			X	Surgery	In pregnancy	N/A	VD
Isla, 1989	1	37	N/A	CT + DSA	N/A	N/A	I	Frontal horn of lateral ventricle	28 weeks	X	X			Surgery	In pregnancy	N/A	N/A
Robinson, 1991	2	N/A	N/A	MRI	N/A	N/A	N/A	N/A	N/A	X				N/A	No	N/A	N/A
		N/A	N/A	MRI	N/A	N/A	N/A	N/A	N/A	X				N/A	No	N/A	N/A
Warner,1996	1	32	N/A	MRI	N/A	N/A	N/A	Chiasm	24 weeks	X				Conservative	No	N/A	CS
Pozzati,1996	2	N/A	N/A	MRI	S	N/A	II	Brainstem	N/A	X	X			Conservative	No	N/A	N/A
		N/A	N/A	MRI	S	N/A	III	Temporal lobe	N/A					Conservative	After delivery	N/A	N/A
Awada,1997	2	27	M	MRI	S	None	II	Temporal lobe	21 weeks	X				ACUTE: Carbamazapine CHRONIC: Carbamazapine	No	N/A	N/A
		26	N	MRI	S	None	II	Temporal lobe	N/A	X				ACUTE: Carbamazepine + Phenytoin CHRONIC: Carbamazapine	No	N/A	N/A
Hoeldtke,1998	1	35	M	CT + MRI	S	Preeclampsia	II	Frontal lobe	24 weeks		X			ACUTE: Diazepam + Phenytoin CHRONIC: Phenytoin	No	33 weeks	VD
Flemming, 2003	1	28	M	MRI	S	None	I	Pons/midbrain	27 weeks	X		X		Surgery	In pregnancy	36 weeks	CS – CCM indication
Safavi Abbasi,2006	1	21	N	MRI	S	None	II	Intramedullary	N/A	X		X		Steroid	2 weeks after delivery	N/A	CS – CCM indication
Haque,2008	1	36	N	MRI	S	None	II	Dorsal pons	N/A			X		Conservative	No	N/A	CS – CCM indication
Aladdin,2008	1	34	N	MRI	S	Depression, Eating disorder, Psychosis	II	Frontal lobe	10 weeks		X			ACUTE: Phenytoin + Loranzepam + Clobazam + Carbamazepine CHRONIC: Phenytoin	No	N/A	Interruption of pregnancy
Ramirez Zamora,2009	1	38	M	MRI	S	None	II	Dorsal pons	N/A			X		Conservative	No	N/A	VD
Cohen Gadol,2009	1	28	N/A	MRI	N/A	N/A	N/A	Pons	29 weeks	X		X		Conservative	yes	Term	CS
Nossek,2011	3	34	N/A	MRI	N/A	N/A	N/A	Brainstem	31 weeks	X	X			Conservative	No	38 weeks	CS
		27	N/A	MRI	N/A	N/A	N/A	Frontal lobe	18 weeks					Conservative	No	40 weeks	VD
		32	N/A	MRI	N/A	N/A	N/A	N/A	11 weeks					Surgery	In pregnancy (11 weeks)	38 weeks	CS
Burkardt,2012	3	31	N/A	MRI	N/A	None	I	Pontomesencephalic	11 weeks	X		X	X	Surgery	In pregnancy (12 weeks)	40 weeks	N/A
		26	N	MRI	N/A	None	II	Pons	III trimester			X	X	Cnservative	3 months after delivery	N/A	N/A
		34	N	MRI	N/A	None	II	Thalamo mesencephalic	N/A	X		X		Conservative	1 week after delivery	N/A	N/A
Ranger,2012	1	15	N	MRI	N/A	None	II	Pontomedullary junction	38 weeks	X		X	X	Conservative	1 month later	38 weeks	CS
Miele, 2012	1	36	N/A	MRI	S	Chiari I	II	Frontal lobe	27 weeks	X		X	X	Surgery	In pregnancy (27 weeks)	Term	CS
Witiw,2012	3	N/A	N/A	MRI	N/A	N/A	N/A	Frontal lobe	I trimester	X				Conservative	No	Term	VD
		N/A	N/A	MRI	N/A	N/A	N/A	Pons	III trimester	X				Conservative	No	Term	CS – CCM indication
		N/A	N/A	MRI	N/A	N/A	N/A	Pons	III trimester	X				Conservative	No	Term	CS – CCM indication
Kalani,2013	4	27	M	MRI	S	None	II	Cervicomedullary junction	N/A		X			N/A	Yes at age 40	Term	VD
		19	M	MRI	F	None	II	Multiple lesions	N/A		X			Conservative	No	Term	VD
		19	M	MRI	F	None	I	Left Insula	N/A	X	X			Antiepileptic	7 weeks after delivery	Term	VD
		28	M	MRI	F	None	II	N/A	N/A		X			N/A	No	Term	CS – CCM indication
Simonazzi,2013	6	N/A	N	MRI	S	N/A	I	Bulb	20 weeks	X		X		Conservative	No	20 weeks	Interruption of pregnancy
		N/A	N	MRI	S	N/A	II	Corpus callosum	33 weeks		X			N/A	No	37 weeks	CS – CCM indication
		N/A	N	MRI	S	N/A	I	Bulb	30 weeks	X		X		Conservative	No	30 weeks	CS
		N/A	N	MRI	S	N/A	I	Pons	36 weeks	X		X		Conservative	6 years from delivery	36 weeks	CS – CCM indication
		N/A	N	MRI	S	N/A	I	Pons	Puerperium	X		X		Conservative	No	40 weeks	CS
		N/A	N	MRI	S	N/A	II	Pons	Puerperium		X			N/A	No	41 weeks	VD
Yamada,2013	1	39	N	MRI	S	N/A	II	Left frontal lobe	I trimester			X	X	Conservative	No	N/A	Interruption of pregnancy
Ulrich,2016	1	34	M	MRI	S	Hashimoto, Osteopenia, Ulcerative colitis, IVF	I	Periaqueductal region of the posterior lateral cerebral peducnle area	21 weeks	X		X	X	Conservative	No	38 weeks	VD
Pars,2016	1	33	N	MRI	S	None	I	Intramedullary	22 weeks			X		Steroids	After delivery	26 weeks	CS
Delanouis, 2017	1	29	N	MRI	S	None	I	Midbrain	5 weeks	X		X		Conservative	2 months after delivery	Abortion	CS-CCM indication
Ya Lan Xu,2017	1	31	M	MRI	S	Thyroid cancer	II	Frontal lobe	36 weeks		X			N/A	No	36 weeks	CS
Hayashi,2017	1	22	N	MRI	S	None	II	Brainstem	38 weeks	X		X		Conservative	No	38 weeks	CS
Bennis,2019	1	32	M	MRI	S	None	III	Epidural	Term			X		Conservative	After delivery	Term	CS
Maier,2019	1	34	N/A	MRI	S	None	I	Pons	24 weeks	X		X		Steroids	No	38 weeks	CS
Saberi, 2021	1	29	M	MRI	S	None	I	Thalamus	34 weeks	X		X	X	Conservative	After delivery	36 weeks	CS-CCM indication
Joseph, 2021	4	27	M	MRI	F	N/A	II	Basal ganglia	III trimester	X				Conservative	No	Term	VD
		38	N	MRI	S	Infertility (IVF)	I	Thalamus	Puerperium	X				Conservative	No	Term	CS
		26	N	MRI	S	N/A	I	Temporal lobe	I trimester	X				Conservative	No	Term	CS
		23	N	MRI	F	N/A	I	Frontal lobe	II trimester	X				Conservative	No	Term	VD
Abougamil, 2023	1	28	M	MRI	S	None	II	Intramedullary conus medullaris	32 weeks			X		Steroids	4 weeks after delivery	36 weeks	CS
Ellwood, 2024	5	36	M	MRI	N/A	N/A	III	N/A	Pre-pregnancy					Conservative	No	40 weeks	CS
		34	M	MRI	N/A	N/A	I	N/A	Pre-pregnancy	X				Conservative	No	40 weeks	CS
		31	M	MRI	N/A	N/A	III	N/A	Pre-pregnancy					Conservative	No	38 weeks	CS
		35	M	MRI	N/A	N/A	III	N/A	Pre-pregnancy					Conservative	No	40 weeks	CS
		33	M	MRI	N/A	N/A	I	N/A	Pre-pregnancy	X				Conservative	No	36 weeks	VD

**Table 2:** Summary table: pregnancy and hemorrhage risk in women with cerebral cavernous malformations in the 3 largest case-control studies

Author, year	Kalani, 2013	Joseph, 2021	Witiw, 2012
Design	Retrospective + prospective registry	Prospective registry	Retrospective cohort with patient survey
Center	Barrow Neurological Institute	Mayo Clinic	University of Toronto
N. of women analyzed	64	90 diagnosed ≤ 45 y	186
Mean age at diagnosis	Not reported	31.6 y (≤ 45 y)	42.7 y
Pregnancies analyzed (total)	168	172	349
Pregnancies after diagnosis	N/A	32	35
Hemorrhagic events during pregnancy	5 (in 4 patients)	0 (after diagnosis)	3 (all antepartum, during pregnancy)
Hemorrhagic events during delivery/postpartum	0	0	0
Total hemorrhagic events (all periods)	N/A	42 during nonpregnant follow-up	49 during childbearing years
Hemorrhage rate per pregnancy	3%	0%	N/A
Hemorrhage rate per patient-year (pregnancy)	3.4%	0%	1.15%
Hemorrhage rate per patient-year (nonpregnancy)	N/A	10.4%	1.01%
Relative risk pregnancy vs. nonpregnancy	NR	N/A	1.13%
Familial vs. sporadic risk per hemorrhagic pregnancies	Familial 4% vs. Sporadic 2%	0%	N/A
Delivery mode (vaginal vs. cesarean)	149 vaginal, 19 C-sections	10 vaginal, 14 C-sections	237 vaginal, 46 C-sections
Hemorrhages during delivery	0	0	0
Seizure as presenting symptom in pregnancy	Yes (4 of 5 hemorrhages)	N/A (no hemorrhages)	Yes (2 of 3 hemorrhages in deep lesions)
Influence on reproductive choices	NR	NR	15 women altered reproductive plans
Conclusions	No significant increase in risk during pregnancy or delivery; vaginal delivery safe	No increase in risk; vaginal delivery appropriate	No increase in risk; vaginal delivery appropriate for obstetrics

### Risk of bias

Study quality was assessed using the JBI tool, for case series and case reports (Tables 1 and 2 **– **Suppl. Material).

### Statistical analysis

Following the systematic review, we performed a meta-analysis of pooled proportions by grouping patients by location, management strategy and Zabramski grade. Statistical heterogeneity was assessed using the I² statistic, with values greater than 50% indicating substantial heterogeneity. All statistical analysis were done using JASP v19.3.0. Chi-square tests were sued for categorical variables and p-values < 0.05 were considered significant. Then, the standardized Pearson residuals (> |1.96|) was utilized to assess the tables with significant associations for statistical significance (Tables 3, 4, and 5).

**Table 3:** Univariate analysis by location type

		Spinal Cord (*n* = 5)	Deep (*n* = 6)	Infratentorial (*n* = 21)	Hemispheric (*n* = 14)	TOTAL (*n* = 46)	*p*-value
Clinical Onset during Pregnancy							
	Hemorrhage	1	6	17	7	31	**0.008**
	Seizure	1	1	3	5	10	0.495
	Headache	0	1	5	2	8	0.626
	Neurological Deficit	4	2	15	2	23	**0.004**
Treatment (*)							**0.048**
	Antiepileptic	0	0	0	5	5	
	Conservative	1	5	16	6	28	
	Surgery	0	1	3	1	5	

Legend: (*): 8 patients excluded for missing data

## Results

The string-search retrieved 312 papers. The first screening excluded 12 non-English-written articles, 23 reviews, 165 studies that did not address the selected population, and 18 articles not pertinent to the topic. The second screening further excluded 62 articles due to insufficient or impertinent data, resulting in 32 studies being included in the final analysis. The overall study selection process was documented using the PRISMA 2020 flowchart, which summarizes the stages of identification, screening, eligibility, and inclusion (Fig. 1; Table 1).

## Systematic review

A total of 56 patients across 32 studies were identified as harboring cerebral or spinal cavernous malformations during pregnancy (Table 1) [1–3, 5–13, 15–18, 20, 22, 23, 25–33, 35–38]. Maternal age was reported in 43 patients, with a mean of 30.8 years (range 15–39 years). Parity status was available in 38 cases, among whom 20 (53%) were multiparous and 18 (47%) were nulliparous [1–3, 5, 6, 8, 10, 11, 13, 15–18, 23, 25, 27, 30–32, 35, 37, 38]. A familial form of cavernomatosis was documented in 5 patients (14,28%) whereas the remaining cases were sporadic or unspecified.

The Zabramski classification of the lesion was described in 45 patients. Among these, Type II cavernomas were the most frequent, reported in 18 cases (32.7%), followed by Type I in 15 patients (27.3%) and Type III in 6 patients (10.9%) [1–3, 5, 6, 8, 9, 12, 13, 15–18, 20, 25, 27, 28, 32, 33, 37]. Gestational age at symptom onset varied: 7 patients became symptomatic in the first trimester, whereas 30 developed symptoms from the second trimester onwards.

Lesion location was available for 41 patients. Brainstem cavernomas accounted for 17 cases (29.3%), including the pons, midbrain, and pontomedullary junction. Supratentorial cavernomas were reported in 18 patients (48.1%), most commonly involving the frontal lobe (9 cases) and temporal lobe (4 cases). Intramedullary spinal cavernomas were described in 3 patients (5.2%). In 9 patients, the precise location was not specified [1–3, 5–13, 15–18, 20, 22, 23, 25–33, 35–38].

Clinical presentation during pregnancy was reported for 56 cases. Forty-four patients presented with bleedings (78.6%), 12 with seizures (21.4%), 23 with neurological deficits (41.1%), and 8 with headaches (14.3%). In 5 cases (8.9%), the lesion was incidentally discovered without acute symptoms [1–3, 5–13, 15, 16, 18, 20, 22, 25, 26, 28, 30–33, 36–38]. Twenty-one patients experienced an uncomplicated pregnancy, while only 1 developed preeclampsia. Concerning risk factors, 9 patients (21%) were > 35 years, no patient were on antiplatelet regimen or had associated venous angiomas (Table 1).

Treatment strategies varied: surgical excision during pregnancy was done in 6 patients (12.2%), while 13 patients (26.53%) underwent surgery in the early postpartum period. Of the patients who underwent surgery during pregnancy, one was operated for sudden onset of vertigo, diplopia, and ataxia [23]; one case underwent preventive surgery as “the risk of rebleeding during gestation had not yet been established in the literature at that time” [15]; one for acute seizure [10]; one based on patient preference [22]; one for life-threatening neurological deterioration associated with acute hemorrhage [6]; and one for acute hemorrhage alone [20]. Conservative management was the most frequent approach, adopted in 32 patients (65.3%). Acute medical treatment included antiepileptic medications (ASMs) in 7 cases and steroid therapy in 4 patients. Pregnancy termination was reported in 4 cases, due to severe hemorrhage or neurological deterioration [1–3, 5–13, 15–18, 20, 22, 23, 25–28, 30–33, 35–38].

Delivery mode was specified for 32 patients. Cesarean section was performed in 19 cases (59.38%), frequently due to cavernoma-related indications or recent neurosurgical intervention (8). Vaginal delivery was achieved in 13 patients (40.6%). In 20 patients, the mode of delivery was not reported [1–3, 5–13, 15–18, 20, 22, 23, 25–33, 35–38].

When stratified by severity of presentation, 42 patients (75%) presented with hemorrhage and/or focal neurological deficits and were therefore classified as severe, whereas 8 patients (14%) presented with seizures alone and were considered mild cases. The remaining cases were incidental or incompletely reported. Among severe cases, management varied: 5 patients underwent surgical resection during pregnancy, while 27 were managed conservatively and nonetheless proceeded safely to delivery, including vaginal delivery in 6/27 patients. In contrast, patients with milder presentations were uniformly managed medically, most often with antiepileptic drugs, and delivered at term without complications.

### Meta-analysis

#### Univariate analysis - Location

Initially, we stratified the dataset by lesion type, specifically spinal, deep (basal ganglia and thalamus), infratentorial and supratentorial (hemispheric) (Table 3). Hemorrhagic episodes during pregnancy were significantly associated with lesion site (*p* = 0.008). Most hemorrhages occurred in infratentorial lesions (17/31; 54.8%), whereas spinal cord lesions rarely bled (1/31; 3.02%), all deep-seated lesions were hemorrhagic (6/31; 19.35%), and half of the supratentorial lesions showed bleeding (7/31; 22.6%).

No significant differences were observed in seizure onset across locations (*p* = 0.495) or in headache occurrence (*p* = 0.626). In contrast, focal neurological deficits were significantly associated with lesion site (*p* = 0.004): they were present in 80% of spinal lesions (4/5), 40% of deep lesions (2/5), 88% of infratentorial lesions (14/16), and 17% of superficial hemispheric lesions (2/12).

Additionally, the type of management varied according to lesion location. Most patients managed conservatively had infratentorial lesions (16/28), followed by superficial (6/28), deep (5/28), and spinal cord (1/28) lesions. Surgical intervention was performed in three infratentorial, one spinal, and one deep lesion, whereas antiepileptic management was used exclusively for five superficial lesions. This difference was statistically significant (*p* = 0.048).

#### Univariate analysis - Zabramski grade

When stratified by Zabramski grade, a significant association with hemorrhage was found (*p* < 0.001), with the majority occurring in grade I lesions (17/18; 94%), compared with grade II (9/22; 41%) and grade III (0/5; 0%). For seizures, the difference was also significant (*p* = 0.037), most commonly affecting grade II lesions (9/11; 81.8%), whereas grade I (2/11; 18.2%) and grade III (0/11; 0.0%) showed fewer cases. No significant differences were observed for focal neurological signs (*p* = 0.241) or headaches (*p* = 0.591) (Table 4).

**Table 4:** Univariate analysis by Zabramski Rage

			I (*n* = 18)	II (*n* = 22)	III (*n* = 5)	TOTAL (*n* = 45)	*p*-value
Clinical Onset During Pregnancy							
		Hemorrhage	17	19	0	26	**< 0.001**
		Seizure	2	9	0	11	**0.037**
		Headache	15	18	5	38	0.591
		Neurological Deficit	11	10	1	22	0.241
Treatment (*)							0.190
	Antiepileptic		1	4	0	5	
	Surgery		12	10	5	27	

Legend: (*): 8 patients were excluded for missing data

#### Univariate analysis – Treatment type

In the end, we stratified data by type of treatment (Table 5). Patients treated with steroids were excluded because of the small sample size (< 5), which violated the Chi-square assumptions. Among the remaining groups, only the occurrence of seizures showed a statistically significant association (*p* = 0.013), with 3 cases in the conservative group, 4 in the antiepileptic group, and 1 in the surgical group.

**Table 5:** Univariate analysis by treatment type

			Conservative (*n* = 34)	Antiepileptic (*n* = 5)	Surgery (*n* = 6)	TOTAL (*n* = 45)	*p*-value
Clinical Onset During Pregnancy							
		Hemorrhage	11	2	1	14	0.673
		Seizure	3	3	1	7	**0.013**
		Headache	29	5	3	37	0.062
		Neurological Deficit	16	0	3	19	0.127
Zabramski Grade (*)							0.255
	I		12	1	3	16	
	II		10	4	1	15	
	III		5	0	0	5	

Legend: (*): 9 patients were excluded due to missing values

## Discussion

Our systematic review shows that cerebral and spinal cavernous malformations in gravid patients are most frequently located in the brainstem and supratentorial regions and tend to present with hemorrhagic events, seizures, or focal deficits. However, the majority of patients in the pooled cohort of case-reports experienced symptomatic presentation during pregnancy, with hemorrhage reported in over 70% of cases [1–3, 5–13, 15–18, 20, 22, 23, 25–33, 35–38]. The meta-analysis showed that most hemorrhagic cases were related to infratentorial or deep-seated malformations (*p* = 0.008). Similarly, neurological deficits were primarily related to spinal cord and infratentorial lesions (*p* = 0.004). However, when analyzing separately the three largest case–control studies [16, 17, 36], accounting for 340 women ad 707 pregnancies, the proportion of hemorrhagic presentations was not increased during pregnancy or the puerperium. In fact, the rate of hemorrhage during pregnancy was consistently low across cohorts (Table 2). In the Kalani study, 5 hemorrhagic cases were identified among 168 pregnancies, corresponding to a 3% hemorrhagic risk, with most events presenting as seizures and occurring antepartum [17]. In 2021, Joseph et al. reported zero hemorrhagic events during pregnancy and delivery, despite capturing 32 pregnancies occurring after CCM diagnosis [16]. Witiw et al. documented three hemorrhages during pregnancy, translating into a hemorrhage rate per pregnancy of approximately 0.9% [36].

Therefore, the apparent overrepresentation of hemorrhagic presentations in the current review is therefore likely due to selective reporting and referral of symptomatic cases.

Nevertheless, the postpartum period is still regarded as a time for caution. General stroke literature shows a peak in ICH risk in the early postpartum period, often related to hypertensive disorders, and some authors advise continued vigilance after delivery [14, 39]. Merlino et al. (2021) explicitly state that the “puerperium is a critical time for the woman with cavernomas” and recommend “ongoing clinical observation” [19]. Bektas et al. (2024) likewise emphasize close monitoring of neurologic symptoms and antiseizure-medication (ASM) levels in the first 4–6 weeks postpartum, as hormonal and metabolic changes continue into this period. This is because it is believed that during pregnancy, increased cardiac output and turbulent blood flow are thought to stress vascular malformations [4]. The abrupt reversal of these conditions after delivery could conceivably impact cavernoma walls, through hormonal withdrawal or changes in coagulation. Additionally, Bektas et al. hypotized that the rise of placental growth factors and hormones during pregnancy and subsequent fall after birth, might transiently alter cavernoma vessel permeability causing bleeding [4]. Moreover, experimental models and some observational studies have demonstrated that estrogen might strengthen endothelial tight junctions, and potentially reduce bleedings [4, 16]. However, the estrogen’s protective effect decreases with age. This, together with increased progesterone signaling, might explain the increased vascular permeability, and increased risk of bleeding seen in older gravids. Nonetheless, cautious interpretation is warranted as these findings derive from retrospective studies, with potential selection and under-reporting bias [16, 17, 36].

Pre-pregnancy counseling remains critical: women should be advised to defer conception for at least a year after a hemorrhagic event, when the risk of recurrence is highest. Additionally, labor, particularly when prolonged, involves significant physical strain that might exacerbate bleeding in an already vulnerable cavernoma [4]. However, epidemiological studies on postpartum hemorrhagic risk factors are lacking, thereby making any discussion purely speculative. Thus, postpartum surveillance remains a precautionary measure, as no quantitative evidence has shown an increase in risk of hemorrhage after delivery [4].

### Management

Our findings also indicate that management strategies remain highly variable and are largely guided by individual clinical circumstances rather than standardized protocols.

Most cases were managed conservatively during pregnancy whenever possible, with definitive surgery deferred until after delivery. Surgical treatment during gestation was generally reserved for patients presenting with hemorrhagic infratentorial lesions, where the neurological risk justified intervention despite pregnancy. Still, the majority of conservatively managed cases involved infratentorial and deep-seated lesions (Table 3, *p* = 0.048), reflecting the increased surgical risk and technical difficulty associated with these locations. Multiple authors recommend delaying nonurgent cavernoma resection until the postpartum period. For example, Merlino et al. state that the only indication for neurosurgery in pregnancy is “rapidly progressive symptoms,” and even then it should be postponed until after 30 weeks or until the puerperium if it arises late in gestation [19]. Gross et al. similarly report that in most series, symptomatic cavernomas presenting during pregnancy were ultimately treated after delivery. In our review, even women who presented with antepartum hemorrhage were often stabilized with medical management, including ASMs if seizures occurred, and then underwent resection weeks or months postpartum [2]. Notably, two patients in Merlino’s review had resections at 1 week and 3 months after uncomplicated delivery, both with good outcomes, illustrating that delayed surgery is generally safe if the patient remains stable.

After a cavernoma hemorrhage, anticonvulsant prophylaxis may be beneficial. In the cases reported in the literature, at least three postpartum patients were empirically treated with antiepileptic drugs to prevent seizures secondary to intracranial hemorrhagic injury [4, 16, 21]. This reflects the fundamental principle of providing appropriate care for patients with hemorrhagic CMs to prevent neurological deterioration **(**Tables 6 and 7).

**Table 6:** Risk of congenital malformations and teratogenic effects of Antiseizure medications

Antiseizure Medication	Risk of Major Congenital Malformation (% among registries)	Most Common Reported Teratogenic Effects
Valproic acid	6.7–10.3%	Neural tube defects, hypospadias, cardiac defects, oral clefts
Phenobarbital	5.5–6.5%	Cardiac defects, oral clefts
Phenytoin	2.9–6.4%	Cardiac defects, digit hypoplasia
Carbamazepine	2.6–5.5%	Neural tube defects
Topiramate	3.9–4.3%	Oral clefts
Oxcarbazepine	2.2–3.0%	No evidence of teratogenicity
Lamotrigine	2.0–2.9%	No evidence of teratogenicity
Levetiracetam	0.65–2.8%	No evidence of teratogenicity

**Table 7:** Effects of Antiseizure medications on bone mineral density and osteoporosis prevention recommendations

Antiseizure Medications and Bone Mineral Density	Recommendations for Osteoporosis Prevention
Lower bone mineral density	
• Benzodiazepine • Carbamazepine • Gabapentin • Oxcarbazepine • Phenobarbital • Phenytoin • Primidone • Valproic acid • Zonisamide	• Monitor calcium and vitamin D levels 1–2 times/year • Daily calcium 1,200 mg + vitamin D 800 IU • Weight-bearing exercise 30 min daily • Tobacco cessation • Moderate/no alcohol and caffeine intake • Avoid glucocorticoids if possible
Possibly lower bone mineral density	
• Levetiracetam • Topiramate	If osteopenia/osteoporosis detected: • Consider bisphosphonates • Consider switching antiseizure medication if relevant
Do not affect bone mineral density	
• Lamotrigine	• Maintain vitamin D target levels > 30 ng/mL

Another critical issue concerns the role of exogenous hormones. A multicentric study by Zuurbier et al. showed that hormone replacement therapies (HRT) and oral contraceptives (OCP) are associated with a higher risk hemorrhage (33.6% vs. 15.6%) [40]. However, the existing literature is yet insufficient to quantify these risks. Whenever possible, the use of estrogen-based contraceptives and oral HRT should be discouraged, particularly in patients with deep-seated lesions and prior hemorrhage. In these cases, copper IUDs or lowest effective hormonal doses could be an alternative [4].

Furthermore, in cases presenting seizures, another issue of concern pertains the interplay between ASMs and hormonal therapies. ASMs reduce contraceptive efficacy, increase the risk of potential exposure to teratogens early in gestation and increase the probability of unwanted pregnancies. At the same time, OCPs lower serum levels of ASMs, thereby impairing seizure control [4, 40]. Nevertheless, roughly 20–30% of women physiologically experience seizure worsening during pregnancy. For this reason, meticulous preconception planning, regular drug level monitoring, and dose modifications throughout pregnancy are essential [4, 40].

Additionally, in our analysis, of the women who presented with seizures, three were treated with ASMs, one underwent surgical treatment and three were managed convervatively (Table 5, *p* = 0.013). This suggests that seizure alone was insufficent to dictate treatment choices. In contrast, topography and bleeding were essential in guiding managment decisions.

Another aspect to take into account when choosing ASMs relates to bone health: women are inherently at higher risk for osteoporosis, and long-term use of ASM may further exacerbates this risk. Lifestyle modification, dietary supplements and bone mineral density screening, are recommended [4].

Finally, mental health should not be overlooked, as patients living with CM often suffer from anxiety, depression, and fatigue. The additional concerns regarding hemorrhagic risks, seizure control and reproductive planning can further amplify psychological distress. Psychological support and rehabilitation programs can be invaluable in improving patients’ quality of life [4].

### Delivery

In all studies, the mode of delivery was guided by the obstetric indications. Our analysis showed that vaginal delivery was redeemed safe, as only rare cases of bleeding were reported [3, 8, 16, 20, 28, 33, 34]. Although most births were through elective cesarean deliveries, our review did not show any benefit in preventing hemorrhage, thereby cesarean was opted solely out of caution. Therefore, in the absence of other concerns, the mode of delivery should not be dictated by the status of the cavernoma [4].

### Pooled case reports vs. case series

The findings of the pooled case reports (Table 1) differ significantly from those reported in the largest observational cohorts (Table 2). The latter consistently suggest that pregnancy is not associated to an increased risk of hemorrhages in women with CCMs and that vaginal delivery is safe. Of note, these cohorts primarily capture longitudinal, population-level risk estimates across all pregnancies and follow-up periods. In contrast, the pool of case reports, likely driven by selective reporting of clinically severe cases, reflects a different pattern. Therefore, the pooled cohort overrepresents high-acuity events and cannot be interpreted as an estimate of baseline hemorrhage risk during pregnancy. Rather, it provides complementary insight into the clinical spectrum, timing, and management of symptomatic cases encountered in real-world practice. Importantly, despite these differences in case mix, our findings remain concordant with large cohort data in showing no excess hemorrhagic risk during delivery and supporting the safety of vaginal delivery in appropriately selected patients.

### Limitations

This systematic review has several important limitations. First, most of the included studies were single-case reports or small case series, carrying an inevitable risk of overrepresentation of complicated scenarios. This explains the overestimated incidence of hemorrhage and neurologic deterioration compared to the three case-control studies. Second, confounding variables such as hypertension, preeclampsia or anticoagulant use were not systematically reported in the included studies. Also, data extraction was often limited by incomplete reporting of key clinical details. Additionally, variability in follow-ups and outcome reporting precluded a meta-analysis of maternal–fetal outcomes. Finally, the lack of prospective studies warrants for a cautious interpretation of these findings. Future studies reporting systemic risk factors could aid in developing a treatment protocol for pregnant women affected by CMs.

## Conclusions

Cavernous malformations in pregnancy are uncommon and, based on available evidence, do not appear to carry a substantially increased risk of hemorrhage compared to the nonpregnant state. Nonetheless, individualized management and heightened vigilance during the peripartum and postpartum periods remain essential to ensure optimal maternal and fetal outcomes.

## Supplementary Information

Below is the link to the electronic supplementary material.

Supplementary file 1 (DOCX 696 KB)

Supplementary file 2 (DOCX 688 KB)

## Data Availability

Available upon reasonable request.

## Abbreviations

CM: Cavernous Malformation
ASM: Antiseizure Medication
ICH: Intracerebral Hemorrhage
MRI: Magnetic Resonance Imaging
IUD: Intrauterine Device
